# The Influence of Age on the Activity of Selected Biochemical Parameters of the Mouflon (*Ovis musimon* L.)

**DOI:** 10.3390/ani9050242

**Published:** 2019-05-15

**Authors:** Terézia Pošiváková, Jozef Švajlenka, Ján Pošivák, Jaroslav Pokorádi, Rudolf Hromada, Peter Korim, Ladislav Molnár

**Affiliations:** 1 Department of the Environment, Veterinary Legislation and Economy, University of Veterinary Medicine and Pharmacy of Košice, Komenského 73, 041 81 Košice, Slovakia; rudolf.hromada@uvlf.sk (R.H.); peter.korim@uvlf.sk (P.K.); 2 Department of Construction Technology and Management, Faculty of Civil Engineering, Technical University of Košice, Vysokoškolská 4, 042 00 Košice, Slovakia; 3 Clinic for ruminants, University of Veterinary Medicine and Pharmacy of Kosice, Komenského 73, 041 81 Košice, Slovak Republic; jan.posivak@uvlf.sk; 4 Xcell Breeding Services Ltd., 81101 Bratislava, Slovakia; jaroslav.pokoradi@xcell.sk; 5 Clinic for birds and exotic animals, University of Veterinary Medicine and Pharmacy of Kosice, Komenského 73, 041 81 Košice, Slovak Republic; ladislav.molnar@uvlf.sk

**Keywords:** biochemical status, biomarker, mouflon, reference interval, wildlife

## Abstract

**Simple Summary:**

Blood analyses can be used to assess the health and physiological conditions of wild animals and may provide a precise picture of disease, habitat quality, and other environmental factors. The objective of this study was to analyze the relationship between the age and selected biochemical parameters of the female mouflon (*Ovis musimon* L.). This study aims to create a possible reference range of biochemical parameter concentrations in the mouflon and to extend the knowledge of this species of wild game within our geographic region.

**Abstract:**

Blood analyses can be used to assess the health and physiological conditions of wild animals and may provide a precise picture of disease, habitat quality, and other environmental factors. The objective of this study was to analyze the relationship between the age and the selected biochemical parameters of the female mouflon (*Ovis musimon* L.). This study creates a possible reference range of biochemical parameter concentrations in mouflon and aims to extend the knowledge of this wild game species within our geographical region. There have not been many studies dealing with this issue in our geographical region. A total of 57 female mouflons aged between 1 and 6 years (1–3 years *n* = 32, 4–6 years *n* = 25) and in good physical condition, with an average live weight between 32 and 40 kg were included in the research experiment between the two years. A total of 15 selected biochemical parameters were analyzed. Using statistical analysis, we noticed significant effects of age on almost three-quarters of analyzed biochemical parameters. A statistically significant correlation was observed between age and the parameters of glucose, alkaline phosphatase, alanine aminotransferase, aspartate aminotransferase, bilirubin, cholesterol, creatinine, high-density lipoprotein, calcium, triglycerides, and urea. An evaluation of the European mouflon’s biochemical parameters during the whole year may be a reliable method for judging a herd’s condition, diagnosing medical disorders, and preventing the etiology of their occurrence. An analysis of biochemical parameters tells us about the functioning of individual organs as well as the animals’ metabolism. Knowledge of the values of blood parameters is of special importance because they allow us to gather more information on mouflons.

## 1. Introduction

An organism’s response to various environmental factors may vary in extent and character, mostly presenting as changes in the animal’s ecophysiological processes and ethology [1,2,3,4,5]. A set of such changes can be studied and monitored using various laboratory examinations. A clinical interpretation of the laboratory examinations of various animal species is an important precondition for correct disease diagnostics, for monitoring animals’ ecophysiological status changes, and subsequently their therapy and individual medical status assessment [6,7,8].

Timely and correct treatment can remove many problems and prevent the further deterioration of an animal’s medical status. In terms of zoocenosis, we see periodic biorhythmic changes determining various exogenous and endogenous rhythms with annual periodicity, where these rhythms are synchronized by annual changes in an organism’s internal and external environment [9,10,11]. The most common examples of circannual rhythms include migration, reproduction tied to a photoperiod in a period during the year, hibernation, shedding fur, the growth of antlers in deer, and also involves natural maturation and old age [12,13]. In the case of many vertebrates, reproduction is tied to a certain age and season [14]. We can observe this in most animals living in the wild, as well as in domesticated farm animals. This process is influenced by age and photoperiod combined with the intense impact of the effect of the length of daylight. Before and during this period, various physiological, morphological, and ethological changes occur in animals [15,16]. Among physiological changes, it is mainly the hormonal activity of endocrine systems that determines overall medical and reproductive status [17]. There are also changes in the concentration of individual biochemical parameters [18,19]. By studying and observing physiological changes in an animal organism and by monitoring the concentration of selected biochemical parameters using suitable laboratory methods, we can determine and judge an animal’s medical status [3]. Changes in an internal environment cause conditions such as the responses of animals to the causes of their diseases, stress, damage or pathogenic conditions caused by microorganisms, parasites, and chemical or physical factors [12,20,21]. Besides these factors, an animal’s medical status is influenced by age, sex, the breed of animal, and seasonality [22,23,24].

Mouflon farming in small isolated groups without a farming objective, quality veterinary check-ups, and the care of animals, or the application of selection principles, leads to the qualitative degeneration of the group and erodes its gene pool [25,26]. The regular monitoring of medical conditions along with the breeding and veterinary check-ups of animals are recommended in wild game [27,28]. This ensures that detailed information on origin, condition, and biometric properties is recorded during the individual’s birth. The obtained information is used in the case of female breeders for their classification in reproduction. Female breeders are selected on the basis of a large physical frame, good condition, timely rut, early birth of young, good milk production, the careful leading of the young, and uniformity of coloration. A comprehensive veterinary check-up in the presence of a veterinarian is also performed. The examination consists of an external assessment of medical condition, examination, possible injuries, deworming, blood samples and droppings for serological and coprological examination, and other types of intervention such as sonographic examination of early pregnancy, in order to improve the reproductive indicators of farmed animals [28,29,30].

This work addresses the problems of the coordinated monitoring of the ecophysiological status of mouflons living in the natural conditions of their environment while taking account of individual age. The results of such coordinated and targeted monitoring point towards the environment’s relative state and also record the condition of animals living in the wild. Expert research and experimental interventions in monitoring the medical status of animals pursue certain defined ecological objectives, namely, preserving a natural balance between wild game and its environment, preserving the natural quality of the animals’ gene pool, the targeted enhancement of the animals’ breeding quality, and the modification of the animals’ state to reach their optimal state. Based on these facts, our research goal was to analyze the relationship between age and the selected biochemical parameters of the female mouflon (*Ovis musimon* L.). These data provide baseline information about serum biochemical parameters that can be used to manage the health of mouflons in our geographical region.

## 2. Materials and Methods

Our study was realized in accordance with the Slovak Animal Welfare Act and The Veterinary Care Act No. 37/2007. Samples were collected when the herd was captured for a routine veterinary inspection. Animal handling was in accordance with these guidelines to ensure their welfare.

### Study Area

Thanks to its diverse natural conditions the Slovak Republic provides rich Central European hunting areas with either indigenous or introduced wild animals. Our analysis of the Slovak Republic’s climate was based on its geographical location in Europe, or Central Europe, and its belonging to the corresponding climate zone and climate area [31,32]. In terms of global climate classification, the Slovak Republic’s territory belongs in the northern moderate zone with regularly changing seasons and changeable weather, with a relatively even distribution of precipitation during the year [33].

The experimental group consisted of 57 female mouflons at the ages of 1 to 6 years, included in the research experiment between the two years of study. Samples were collected in winter when the herd was captured for a routine veterinary inspection. The average live weight of the members of the female groups was 32–40 kg. The animals’ live weights were measured after blood sampling and were collected by a portable hanging weight machine with a digital display. The mouflons used in this research came from a game reserve in the eastern part of the Slovak Republic at an altitude of 224 m a.s.l. The climatic area in question is characterized by a warm lowland climate with long, warm, and dry summers and short, cold, and dry winters with little snow cover. The forest biotope is characterized by an average structure and species composition of predominantly deciduous trees. Water intake from natural water sources was unrestricted, and the animals were fed hay during winter. The animals showed no signs of disease. During our study, the animals were rounded up using a standard catcher device in the morning. The animals were then caught by hand. This is considered as a more suitable technique for this type of ungulate, as it is thought to cause little stress to the animals. Blood samples were taken from the jugular vein by a veterinarian. Samples were collected once from each animal by blood collection tubes (Serum-SST TM II Advance, BD Diagnostics, Franklin Lakes, NJ, USA) and heparin tubes (Heparin a PST TM II + gel, BD Diagnostics) then immediately centrifuged. Blood serum was stored at −20 °C until analyzed. The following biochemical variables were analyzed: albumin (ALB), alkaline phosphatase (ALP), alanine aminotransferase (ALT), aspartate aminotransferase (AST), lactate dehydrogenase (LDH), glucose (GLU), bilirubin (BILTS), calcium (Ca), phosphorus (P), cholesterol (CHOL), high-density lipoprotein (HDL), low-density lipoprotein (LDL), triglycerides (TRIGL), creatinine (CREA), and urea (UREA). Biochemical parameters were analyzed in relation to animal age using a modern COBAS^®^ c111 biochemical analyzer (Roche, Switzerland) with a flexible system for consolidating routine examinations for various types of biological materials measured with one of four different technologies, namely, absorption photometry, turbidimetry, fluorescence polarization, or ion-selective potentiometry [34]. The obtained results were statistically processed using Statistica 12 (StatSoft, Inc., Tulsa, OK, USA) by means of the Spearman correlation coefficient and the Unpaired *t*-test. A graphical instrument (a point grapher) was chosen for an initial examination of the relation between the ages of the animals and the selected biochemical parameters. Using a correlation analysis, we determined the rate of correlation intensity between two numerical variables (i.e., between age and the selected biochemical parameters) [35]. For a more detailed analysis, the data were divided into two groups by age. The first group (Young) consisted of individuals from 1 to 3 years old, and the second group (Adult) consisted of individuals from 4 to 6 years old. An unpaired *t*-test was used to compare the Young and Adult animal groups.

## 3. Results

The rate of correlation intensity between the ages of the individuals and the selected biochemical parameters was determined using the Spearman correlation coefficient. The summarized results are shown in Table 1.

**Figure 1:**
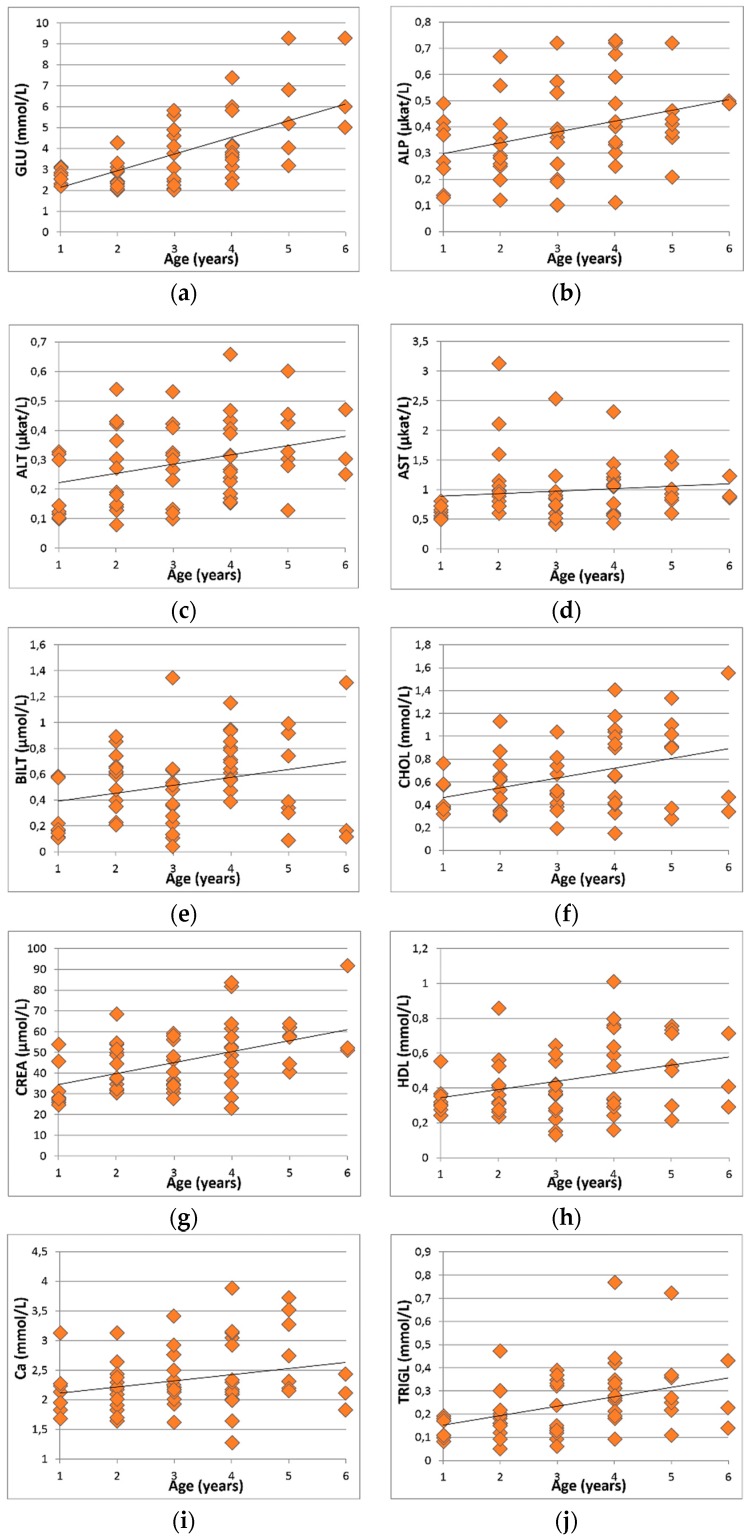

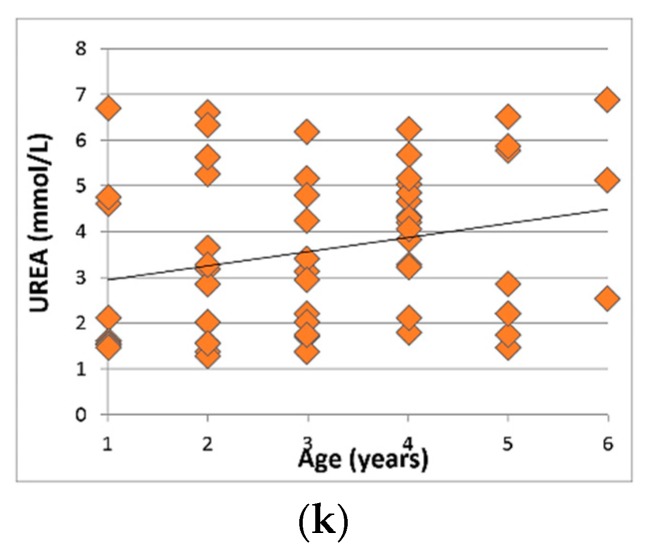
The influence of age on (**a**) Glucose (GLU); (**b**) Alkaline phosphatase (ALP); (**c**) Alanine aminotransferase (ALT); (**d**) Alkaline phosphatase (AST); (**e**) Alkaline phosphatase (BILT); (**f**) Cholesterol (CHOL); (**g**) Creatinine (CREA); (**h**) High-density lipoprotein (HDL); (**i**) Calcium (Ca); (**j**) Triglycerides (TRIGL); (**k**) Urea (UREA).

Based on our statistical analysis, we did not record the statistically significant influence of age on the parameters ALB, P, LDH, or LDL. We observed the following statistically significant correlations at the level of significance of *p* < 0.05 between age and ALT (r = 0.3227), AST (r = 0.2836), BILT (r = 0.2602), Ca (r = 0.2593), CHOL (r = 0.3060), HDL (r = 0.2855), and UREA (r = 0.2721). Similarly, we observed statistically significant correlations at the level of significance of *p* < 0.01 between age and the parameters ALP (r = 0.3753), CREA (r = 0.4692), and TRIGL (r = 0.4442). For the parameter GLU (r = 0.6434), we recorded a statistically significant correlation between age at the significance level of *p* < 0.0001. For the decisive standard values approaching the reference values, we considered the 25–75 percentile interval listed in Table 1 because this interval selects the extreme values of the total analyzed data set. The statistically significant correlations between the assessed parameters and age are shown in Figure 1a–k.

Based on the comparison shown in Table 2, a statistically significant difference between the Young and Adult animal groups was confirmed. For all of the evaluated parameters, the values in the Adult group were higher than those of the Young group. The most significant difference was observed for GLU (*p* ≤ 0.0001), CHOL (*p* = 0.0009), CREA (*p* = 0.0004), HDL (*p* = 0.0009), and TRIGL (*p* = 0.0006). These findings corroborated the correlation analysis shown in Table 1. Different observations in the statistical group analysis and the correlation analysis were found in only two parameters—AST and LDH.

## 4. Discussion

In wild ruminants as in small ruminants, ALP is found in the liver, bones, intestines, kidneys, placenta, and leukocytes, and therefore its wide reference range—which is larger than other species—has a lower diagnostic value [36]. Generally, the parameter values ALB, ALT, AST, and TBIL in females are affected by the rutting period, during which the values of intermediate metabolism in terms of enzyme reduction are changed [17,37,38]. However, ALT is a non-specific enzyme because it is found in muscles and mainly in the liver. Therefore, in ruminants it has a low diagnostic value [36]. The parameter values of CREA, LDH, and GLU are mainly affected by stress during manipulation and consequently increased muscle activity [39,40]. The mechanisms that affect Ca are complex and involve interaction with other chemicals (i.e., proteins and hormones). CHOL level is mainly dependent on nutrition, but some authors [40] describe the elevated level of total CHOL in the first phase of short-term stress in wild ruminants. CHOL is mainly influenced by the income and expenditure of energy stimulants. Phosphorus has an important role in the management of metabolism and is a component of nucleic acids, phospholipids, and nucleotides. TRIGL are synthesized in the liver, specifically from carbohydrates, deposited in fat tissues, and are a secondary source of energy. UREA levels are the main indicator of liver status. UREA levels mainly affect the supply of fluids [38,40].

There have not been many studies on biochemical variables in wild mouflons in the geographical region of Central Europe. This study constitutes an assessment of the biochemical status of selected biochemical variables as influenced by mouflons’ age.

Ciberej et al. [41] analyzed the biochemical status of the mouflon (*Ovis musimon*). The average values of GLU (3.839 ± 1.696 mmol/L), ALB (23.713 ± 8.578 g/L), ALP (0.386 ± 0.169 µkat/L), CREA (45.731 ± 15.347 µmol/L), TRIGL (0.239 ± 0.140 mmol/L), and UREA (3.595 ± 1.725 mmol/L) we measured for female mouflons were lower than the average values of GLU (6.74 ± 4.06 mmol/L), ALB (67.85 ± 80.97 g/L), ALP (3.38 ± 2.00 µkat/L), CREA (141.97 ± 23.66 µmol/L), TRIGL (0.42 ± 0.16 mmol/L), and UREA (6.84 ± 1.83 mmol/L) that Ciberej et al. measured for females. In the case of AST (1.16 ± 0.75 µkat/L) and ALT (0.29 ± 0.08 µkat/L) in females, they recorded findings similar to those we recorded for AST (0.981 ± 0.505 µkat/L) and ALT (0.289 ± 0.137 µkat/L). Didara et al. [42] were concerned with monitoring biochemical parameters in mouflons (*Ovis orientalis musimon*), according to age and sex, living in Croatia (Southern Europe), studying biochemical parameters similar to ours. These authors analyzed 22 adult males and 19 adult females, some of which were pregnant. Similar to our study, the monitoring was conducted during winter. These authors recorded similar average values of TRIGL (0.25–0.26 mmol/L) in female mouflons. They also recorded average values of ALT in females (0.28 µkat/L) that were similar to those in our study. In the case of other analyzed parameters in males, we found differences compared to these authors in the case of ALB, AST, BILTS, CREA, LDH, GLU, ALP, CHOL, HDL, LDL, Ca, P, and UREA parameters, which were markedly higher in these authors’ work than in our findings. These comparisons show that the compared mouflons in Southern Europe had different values of the studied biochemical parameters than the mouflons from our Central European region. It is also interesting to point out that the animals studied by the aforementioned authors came from our region of Slovakia (Central Europe). These authors’ results corresponded with our findings, pointing towards relations between the biological status of females and age. These claims were matched by the research by Kiran et al. [43], who analyzed small ruminants from Southern Punjab in Pakistan. Marko et al. [44] were concerned with the hematological and biochemical parameters of the European mouflon. The average values of ALB (26.3 ± 7.3 g/L), CHOL (0.95 ± 0.29 mmol/L), CREA (84.0 ± 14.1 µmol/L), GLU (3.69 ± 1.66 mmol/L), Ca (2.27 ± 0.36 mmol/L), and P (2.35 ± 0.55 mmol/L) of the females measured by Kiran et al. were higher than those we recorded for ALB (23.713 ± 8.578 g/L), CHOL (0.645 ± 0.318 mmol/L), CREA (45.731 ± 15.347 µmol/L), GLU (3.839 ± 1.696 mmol/L), Ca (2.334 ± 0.554 mmol/L), and P (1.398 ± 0.747 mmol/L) in females. Peinado et al. [45] were concerned with the biochemical parameters of wild mouflon (*Ovis musimon*). The average values of TRIGL (0.83 ± 0.49 mmol/L) and AST (1.716 ± 0.74 µkat/L) measured by Peinado et al. were higher than our recorded average values of TRIGL (0.239 ± 0.140 mmol/L) and AST (0.981 ± 0.505 µkat/L). Mostaghni et al. [46] analyzed the hematological and biochemical parameters of mouflons living in the wild in Iran. The average values of CHOL (0.98 ± 0.53 mmol/L) measured by Mostaghni et al. were higher than our average values of CHOL (0.645 ± 0.318 mmol/L).

Based on a comparison with similar work concerned with monitoring the biochemical status of mouflon and related species of animals, we recorded a variety of differences, which we attributed mainly to the different climatic and geographical conditions in which the examined individuals lived. Other factors such as physical condition, nutrition, stress factors, and the overall environment certainly bore upon the different findings [22,23,24]. It is precisely for this reason that in the future we wish to focus on monitoring other factors that affect the overall health status of the given species of animals. Since the mouflon (*Ovis musimon* L.) has not been sufficiently surveyed in our geographical region, this study contributed to its understanding.

## 5. Conclusions

We recorded a correlation between the age and almost seventy-five percent of the considered parameters through statistical analysis. At the level of significance of *p* < 0.05 we found statistically significant correlations between age and the following parameters: ALT, AST, BILT, Ca, CHOL, HDL, and UREA. At the level of significance (*p* < 0.01) have been observed correlations with the parameters ALP, CREA, and TRIGL. Even with GLU, we recorded a statistically significant correlation influenced by age at the significance level of *p* < 0.0001. The analysis of the Young and Adult groups also confirmed a statistically significant difference between the studied animal groups for almost all parameters assessed. Using analytical processing and analyses of the collected data, we found a significant impact of age on the selected biochemical parameters of mouflon, which affect their overall ecophysiological status. This work provides a scientific research contribution and the results can be used and applied in practice. In order to maintain the stability of the animals’ internal environment—especially in game reserves and on farms, where animals do not have a full range of suitable food—monitoring macro- and microelements is necessary for providing the amounts sufficient for the ecophysiological activity of organs and the proper conversion of nutrients. By comparing our findings with other authors, we recorded differences in the biochemical statuses of the monitored animals, which can be attributed to several factors. As there has been no previous report describing important biochemical variables of mouflon (*Ovis musimon* L.) in Slovakia, the data in our study can be used as guidelines for future diagnosis and research. Since the experimental group of animals were healthy and in optimal body condition, the acquired data can be used as reference intervals for the investigated species and observed categories in our geographical locations.

## Figures and Tables

**Table 1:** The correlation between the ages and the activity of the selected biochemical parameters of mouflon females.

Parameters	x ± std (*n* = 57)	25–75 Percentile	Spearman Correlation Coefficient
GLU (mmol/L)	3.839 ± 1.696	2.46–4.835	0.6434 ***
ALB (g/L)	23.713 ± 8.578	17.15–28.78	ns
ALP (µkat/L)	0.386 ± 0.169	0.262–0.490	0.3753 **
ALT (µkat/L)	0.289 ± 0.137	0.153–0.408	0.3227 *
AST (µkat/L)	0.981 ± 0.505	0.624–1.138	0.2836 *
BILT (µmol/L)	0.523 ± 0.307	0.240–0.705	0.2602 *
Ca (mmol/L)	2.334 ± 0.554	1.982–2.602	0.2593 *
CHOL (mmol/L)	0.645 ± 0.318	0.380–0.900	0.3060 *
CREA (µmol/L)	45.731 ± 15.347	32.210–56.872	0.4692 **
HDL (mmol/L)	0.443 ± 0.201	0.292–0.582	0.2855 *
LDH (µkat/L)	6.51 ± 2.73	4.16–8.82	ns
LDL (mmol/L)	0.151 ± 0.126	0.080–0.187	ns
P (mmol/L)	1.398 ± 0.747	0.772–2.147	ns
TRIGL (mmol/L)	0.239 ± 0.140	0.140–0.317	0.4442 **
UREA (mmol/L)	3.595 ± 1.725	2.010–5.095	0.2721 *

Note: ALB—albumin; ALP—alkaline phosphatase; ALT—alanine aminotransferase; AST—aspartate aminotransferase; LDH—lactate dehydrogenase; GLU—glucose; BILTS—bilirubin; Ca—calcium; P—phosphorus; CHOL—cholesterol; HDL—high-density lipoprotein; LDL—low-density lipoprotein; TRIGL—triglycerides; CREA—creatinine; UREA—urea; x—average value; std—standard deviation; ns—not significant; ***—extremely significant (*p* < 0.0001); **—very significant (*p* < 0.01); *—significant (*p* < 0.05).

**Table 2:** A comparison of the age groups of the animals in terms of the selected biochemical parameters.

Parameters	Young (1–3 Years) (*n* = 32)	Adult (4–6 Years) (*n* = 25)	Unpaired *t*-Test
	x ± std	x ± std	
GLU (mmol/L)	3.082 ± 1.070	4.897 ± 1.889	<0.0001 ***
ALB (g/L)	23.368 ± 6.741	24.197 ± 10.917	ns
ALP (µkat/L)	0.332 ± 0.152	0.461 ± 0.169	0.0015 **
ALT (µkat/L)	0.262 ± 0.134	0.327 ± 0.137	0.0354 *
AST (µkat/L)	0.939 ± 0.578	1.041 ± 0.397	ns
BILT (µmol/L)	0.439 ± 0.280	0.641 ± 0.317	0.0057 **
Ca (mmol/L)	2.219 ± 0.439	2.494 ± 0.669	0.0301 *
CHOL (mmol/L)	0.539 ± 0.211	0.794 ± 0.389	0.0009 ***
CREA (µmol/L)	40.268 ± 11.551	53.379 ± 17.186	0.0004 ***
HDL (mmol/L)	0.375 ± 0.147	0.538 ± 0.234	0.0009 ***
LDH (µkat/L)	5.99 ± 2.52	7.24 ± 2.92	0.0405 *
LDL (mmol/L)	0.149 ± 0.152	0.154 ± 0.083	ns
P (mmol/L)	1.263 ± 0.653	1.587 ± 0.853	ns
TRIGL (mmol/L)	0.191 ± 0.102	0.306 ± 0.162	0.0006 ***
UREA (mmol/L)	3.197 ± 1.749	4.154 ± 1.596	0.0172 *

Note: ALB—albumin; ALP—alkaline phosphatase; ALT—alanine aminotransferase; AST—aspartate aminotransferase; LDH—lactate dehydrogenase; GLU—glucose; BILTS—bilirubin; Ca—calcium; P—phosphorus; CHOL—cholesterol; HDL—high-density lipoprotein; LDL—low-density lipoprotein; TRIGL—triglycerides; CREA—creatinine; UREA—urea; x—average value; std—standard deviation; ns—not significant; ***—extremely significant (*p* < 0.0001); **—very significant (*p* < 0.01); *—significant (*p* < 0.05).
